# Continuous In Vivo Monitoring of Indole-3-Acetic Acid and Salicylic Acid in Tomato Leaf Veins Based on an Electrochemical Microsensor

**DOI:** 10.3390/bios13121002

**Published:** 2023-11-28

**Authors:** Lingjuan Tang, Daodong Li, Wei Liu, Yafang Sun, Ying Dai, Wenjing Cui, Xinliu Geng, Dayong Li, Fengming Song, Lijun Sun

**Affiliations:** 1 School of Life Sciences, Nantong University, Nantong 226019, China; tlj0926@ntu.edu.cn (L.T.); 2008310015@stmail.ntu.edu.cn (D.L.); 2209310015@stmail.ntu.edu.cn (W.L.); 2109110183@stmail.ntu.edu.cn (Y.S.); 2109110167@stmail.ntu.edu.cn (Y.D.); 2109110166@stmail.ntu.edu.cn (W.C.); 2109110218@stmail.ntu.edu.cn (X.G.); 2 Analysis and Testing Center, Nantong University, Nantong 226019, China; 3 National Key Laboratory for Rice Biology, Institute of Biotechnology, Zhejiang University, Hangzhou 310029, China; dyli@zju.edu.cn (D.L.); fmsong@zju.edu.cn (F.S.)

**Keywords:** indole-3-acetic acid, salicylic acid, electrochemical microsensor, continuous in vivo monitoring

## Abstract

Indole-3-acetic acid (IAA) and salicylic acid (SA), as critical plant hormones, are involved in multiple physiological regulatory processes of plants. Simultaneous and continuous in vivo detection of IAA and SA will help clarify the mechanisms of their regulation and crosstalk. First, this study reports the development and application of an electrochemical microsensor for simultaneous and continuous in vivo detection of IAA and SA. This electrochemical microsensor system consisted of a tip (length, 2 mm) of platinum wire (diameter, 0.1 mm) modified with carbon cement and multi-walled carbon nanotubes, an untreated tip (length, 2 mm) of platinum wire (diameter, 0.1 mm), as well as a tip (length, 2 mm) of Ag/AgCl wire (diameter, 0.1 mm). It was capable of detecting IAA in the level ranging from 0.1 to 30 µM and SA ranging from 0.1 to 50 µM based on the differential pulse voltammetry or amperometric i-t., respectively. The dynamics of IAA and SA levels in tomato leaf veins under high salinity stress were continuously detected in vivo, and very little damage occurred. Compared to conventional detection methods, the constructed microsensor is not only suitable for continuously detecting IAA and SA in microscopic plant tissue in vivo, it also reduces the damage done to plants during the detection. More importantly, the continuous and dynamic changes in IAA and SA data obtained in stiu through this system not only can help clarify the interaction mechanisms of IAA and SA in plants, it also helps to evaluate the health status of plants, which will promote the development of basic research in botany and precision agriculture.

## 1. Introduction

Plant hormones are fundamental signaling molecules that play critical regulatory roles in the development and stress responses of plants [1,2,3,4,5,6]. These hormones include salicylic acid (SA), auxins (primarily indole-3-acetic acid, IAA), cytokinins, abscisic acid (ABA), jasmonates, and others [1,2,3,4,5,6]. Among these hormones, SA is an important phytohormone that regulates defense responses [7,8] and many other physiological processes of plants [9,10]. IAA is particularly crucial due to its significant effects on plant growth; it plays a regulatory role in apical dominance, hypocotyl elongation, lateral root formation, and lateral shoot branching, among other phenomena [11,12,13,14]. Accumulating evidence suggests that the crosstalk between SA and IAA has regulatory impacts on plant development and stress responses [5,6,12,13,14,15,16]. For example, excessive accretion of SA in plants often exhibits phenotypes similar to those of IAA deficiency, while SA deficiency results in high levels of IAA [17,18]. Recent reports demonstrate that SA regulates the synthesis and transport of IAA, thereby affecting root meristem pattern formation [10]. Moreover, IAA antagonizes SA signal transduction to control bacterial infection via the regulating development of the lateral root [19]. It is of importance to note that both IAA and SA can be produced through the shikimate pathway, where shikimate is transformed into chorismate under the action of chorismatesynthase, which is then converted into tryptophan for IAA synthesis or isochorismate for SA synthesis [20]. During the defense responses in plants, an increased concentration of SA might restrict the resources required for plant development by affecting IAA biosynthesis and vice versa. This indicates that plants can fine-tune their defense and growth costs by regulating SA and IAA contents. The level of SA in plants can always be induced by stress, while IAA can be inhibited [5,6]; this can be used to reflect the health stage of the plants. Therefore, due to the important roles of SA and IAA in plants, it is necessary to obtain their dynamic information in situ.

Quantifying SA and IAA in plants is challenging because of their physical and chemical instability. Traditional methods for detecting SA and IAA, including gas or liquid chromatography–mass spectrometry (GC-MS or LC-MS) [21], are mostly limited due to the complex and time-consuming preprocessing steps involved, such as freezing, crushing, sample extraction, and purification, which result in a loss of these small molecules and lower yields. Moreover, the large amount of plant tissue required for these methods and their long duration make it difficult to obtain accurate information in a timely manner. Electrochemical detection offers advantages, such as rapid response speed, high sensitivity, and small volume, making it suitable for accurate detection in situ or in real-time [22,23,24]. Our research group previously constructed disposable flat electrodes using nano material-modified conductive carbon tape combined with a paper-based electrochemical analysis device to detect SA or/and IAA, either in situ or through micro-sampling [25,26,27]. However, the use of electrodes with a diameter of 4 mm for in situ/micro-sampling detection requires plant tissues with a large cross-sectional area, resulting in significant damage to the plants. More important, there has not been a good method to continuously detect the SA or IAA in plants previously. Based on our previous study, the disposable microsensor prepared using the nano-Au modified stainless steel Wire (0.1 mm) could be employed to perform continuous detection of H_2_O_2_ in situ and reducing plant injury; this provided a novel strategy for continuous detection of the signal molecules in the plant [28].

Here, a novel electrochemical microsensor based on the platinum wire (diameter, 0.1 mm), modified with carbon cement and multi-walled carbon nanotubes, was constructed for detection of SA and IAA in the plant’s microzone with minimal plant injury. This microsensor allows in vivo and continuous monitoring of plant hormones in smaller plant tissues. This study provides an effective method for studying the regulatory function of SA and IAA in plants and, thus, paves the way for further research into their crosstalk and modulation, as well as the development of precision agriculture.

## 2. Materials and Methods

### 2.1. Chemicals and Materials

We sourced the necessary materials from various suppliers. The silver(Ag) wire (diameter, 0.1 mm), and platinum (Pt) wire (diameter, 0.1 mm) were purchased from Alfa Aesar Chemical Co., Ltd. (Shanghai, China), while the 0.3-mm capillary was purchased from Shanghai Dongming Glass Instrument Co., Ltd. (Shanghai, China). The 3M superglue used in this study was provided by 3M China Co., Ltd. (Shanghai, China), and the double-conductor copper foil tape was acquired from Shenzhen Wangxing Tape Co., Ltd. (Shenzhen, China). Additionally, we obtained conductive carbon cement and its associated dilution solution from Plano GmbH (Wetzlar, Germany), and a 2% dispersion of MWCNTs with a length of 10–20 µm and internal and external diameters of 5–15 nm and >50 nm, respectively, from Nanjing Xianfeng Nanomaterials Co., Ltd. (Nanjing, China). The tomato seeds used in this study (Shanghai 906) were acquired from Lintong Changfeng Vegetable Breeding Farm (Xi’an, China). Moreover, all other chemicals applied were of analytical grade. In addition, double-distilled water was used during the experimental process.

### 2.2. Material Preparation

To prepare the conductive carbon cement, a dilution solution was used in an equal amount. A 2% solution of multi-walled carbon nanotubes (MWCNTs) was dispersed in water at a concentration of 0.01%. Both SA and IAA were separately dissolved in ethanol at a concentration of 0.1 M and diluted with 0.2 M PBS (pH 7.0) prior to their detection analysis. A mixture of perlite, vermiculite, and plantash (1:6:2) was added to tomato seedlings grown at 22 °C under 8 h/16 h dark/light cycles. To investigate the dynamics of SA and IAA in tomato leaf veins, four-week-old tomato seedlings were treated with water (Control) or 0.3 M Nacl solution (high salinity stress) through root-irrigation, respectively. Each treated plant was maintained in a highly humid growth chamber, and the SA and IAA within tomato leaf veins were detected at different time points after inoculation.

### 2.3. Preparation of Electrochemical Microsensor

**Scheme 1:**
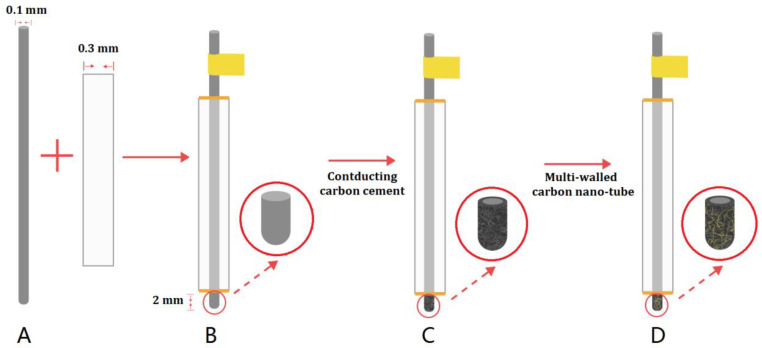
The preparation process of the electrochemical microsensor. (**A**) 0.1 mm bare Pt electrode; (**B**) the bare Pt electrode fixed in capillary; (**C**) the conducting carbon cement (CCC) modified bare Pt electrode; (**D**) the Multi-walled carbon nano-tube(MWCNTS)/conducting carbon cement modified bare Pt electrode (MWCNTS/CCC/Pt).

The Pt wires, each with a length of 50 mm, underwent ultrasonic cleaning for 10 min using ethanol and water. Afterward, each cleaned Pt wire was adhered to a capillary using 3M super glue (Scheme 1A). One end of the wire, with a 2 mm exposure, was used to test samples, while the other end, with a 13mm exposure, was connected to the electrochemical workstation using copper conductive tape (Scheme 1B). The tip (length, 2 mm) of Pt wires were then immersed in conducting carbon cement solution for 10 min and allowed to dry (Scheme 1C). Next, the cement-modified tips of Pt wires were immersed in a 0.01% MWCNTS solution for 10 min, followed by drying (Scheme 1D). This modification produced a working electrode composed of the Pt wire, carbon cement, and multi-walled carbon nanotubes. The Pt wire and silver wire were fixed separately as the Pt wire. The fixed tip (length, 2 mm) of Pt wire was serving as the counter electrode. In addition, the fixed tip (length, 2 mm) of silver wire served as the reference electrode (Ag/AgCl), which was immersed in disinfectant.

### 2.4. Electrochemical Measurements

The electrochemical measurements were performed using the CHI660E electrochemical station. The electrochemical microsensor detection system was utilized for IAA and SA detection through differential pulse voltammetry (DPV) with a potential scope of 0–1 V, increasing potential of 10 mV, amplitude of 50 mV, pulse width of 0.02 s, sample width of 0.0067 s, plus period of 0.5 s, and quiet time of 2 s. To enable continuous detection of SA or IAA, amperometric I-t curves were used; the parameter settings of the amperometric I-t are the original potential of 0.75 (SA)or 0.5 V (IAA), a running time of 3600 s, a sampling interval of 0.1 s, quiet time of 2 s, and scale number of 1 during the run.

### 2.5. qRT-PCR Analysis of Gene Expression

To evaluate the changes in SA and IAA within the tomato leaf veins, the levels of SLNPR1 and SLIAA1 were explored. Total RNA extraction from the tomato leaf veins was performed using TRIzol reagent (Invitrogen, Shanghai, China) followed by purification with the use of RNase-free DNase (TaKaRa, Dalian, China). AMV reserve transcriptase (TaKaRa, Dalian, China) was used to synthesize the cDNA as per the specific protocols. The synthesized cDNAs were adopted for the analysis of gene expression. The qRT-PCR mixture (25 µL) contained 2 X SYBR premix Ex TaqTM (12.5 µL, TaKaRa, Dalian, China), 7.5 pmol of primers (Table 1), and 0.1 µg of the cDNA. The Roche LC480 qPCR analytic system was adopted for qRT-PCR. The gene expression level was identified by the 2^–ΔΔCT^ approach based on the previous description [29]. Each assay was performed in triplicate.

## 3. Results and Discussion

### 3.1. Characterization of theModified Electrodes

**Figure 1:**
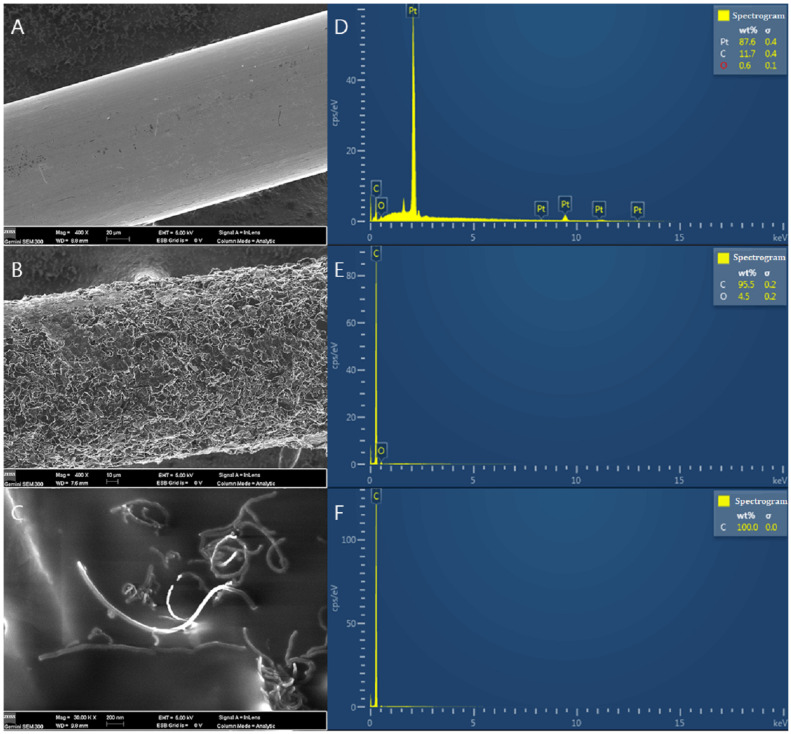
Scanning electron microscope (SEM) analyses (**A**–**C**) and energy dispersive spectrometer (EDS) analyses (**D**–**F**) were conducted on the bare Pt wire, the conducting carbon cement-modified bare Pt wire (CCC/Pt), and the MWCNTS/conducting carbon cement-modified bare Pt wire(MWCNTS/CCC/Pt).

The surface characteristics of the electrodes were explored using a scanning electron microscope ZEISS GeminiSEM 300 (Oberkochen, Germany). As Figure 1A shows, the bare Pt wire had a smooth surface and a diameter of 0.1 mm. After immersion in the conductive carbon cement, the smooth surface of bare Pt wire became rough and uneven, suggesting that the carbon cement covered the surface of the bare Pt wire (Figure 1B). Figure 1B also shows the modified Pt wire with a diameter of approximately 0.1 mm. Figure 1C shows that the conductive carbon cement-modified Pt wire-based electrode surface had nanotubes distributed onto it after immersion in the MWCNTs solution. To analyze the elemental compositions of the modified Pt wire-based electrodes, an energy dispersive spectrometer (Oxford Instruments, Abingdon, UK) was used. The results revealed that Pt was the major constituent in the bare Pt wire, accounting for over 87.6% (Figure 1D), while the contents of C and O elements were approximately 11.7% and 0.6%, respectively. After the Pt wire was modified with the conductive carbon cement, the O and C contents increased to 95.5% and 4.5%, respectively, indicating that the Pt wire surface was covered by the conductive carbon cement (Figure 1E).The presence of 100% C element suggested that the multi-walled carbon nanotubes were also modified on the surface of the modified Pt wire-based electrodes (Figure 1F). These results suggested that the carbon cement and multi-walled carbon nanotubes were successfully modified on the surface of the barePt wire to form the working electrode.

### 3.2. Performance of the Electrochemical Microsensor for Detecting IAA and SA

**Figure 2:**
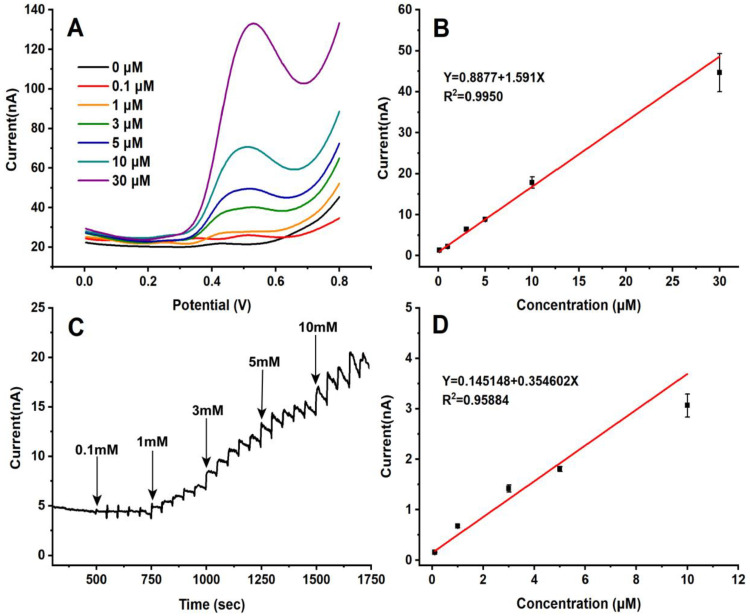
(**A**) DPV detection curves for different IAA contents obtained using the MWCNTS/CCC/Pt wire-based electrochemical microsensor. (**B**) Linear correlation between IAA concentrations and peak currents ranging from 0.1 to 30 µM (*n* = 10). (**C**) Amperometric I-t detection curves for successive additions of 10 µL of IAA at ascending concentrations of 0.1, 1, 3, 5, 10 mM, respectively, in 10 mL of 0.2 M PBS with pH 7 under the application potential of 0.5 V. (**D**) Linearity of peak currents with variations in IAA content from 0.1 to 10 µM (*n* = 10).

**Figure 3:**
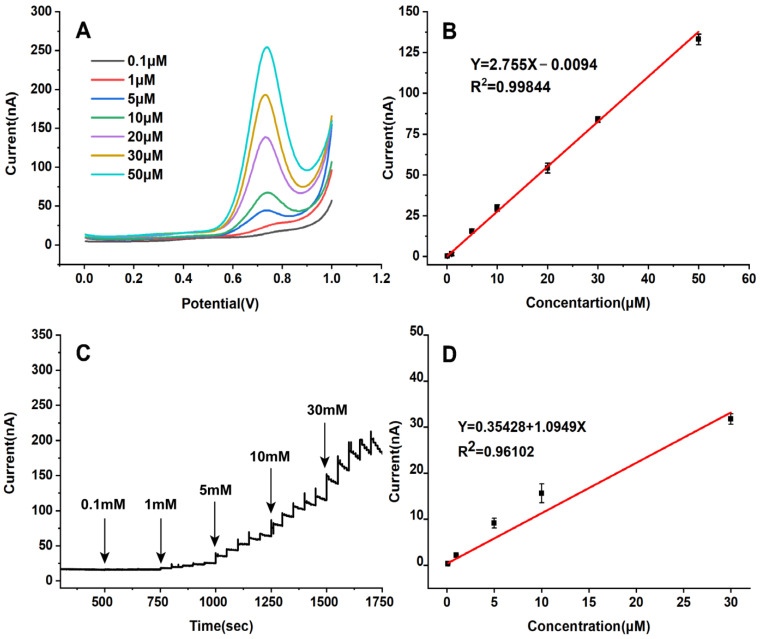
(**A**) DPV detection curves for different SA contents obtained using the MWCNTS/CCC/Pt wire-based electrochemical microsensor. (**B**) Linear correlation between SA concentrations and peak currents ranging from 0.1 to 50 µM (*n* = 10). (**C**) Amperometric I-t detection curves for successive additions of 10 µL of SA at ascending concentrations of 0.1, 1, 5, 10, 30 mM, respectively, in 10 mL of 0.2 M PBS with pH 7 under the application potential of 0.75 V. (**D**) Linearity of peak currents with variations in SA content from 0.1 to 30 µM (*n* = 10).

The electrochemical microsensor utilized DPV to measure the IAA and SA content. In Figure 2A, IAA content was determined based on its peak DPV potential (0.5 V) on the microsensor in the 0.2 M PBS buffer (pH 7.0). As the IAA concentration increased, the DPV responses also increased accordingly. The current variation was found to be linearly related to the IAA concentration at 0.1–30 µM (Figure 2B). SA content was similarly determined based on its peak DPV potential (0.75 V) using the same detection system (Figure 3A), and the variation in current was linearly related to SA content at 0.1–50 µM (Figure 3B). As the same detection system had different DPV potential of IAA and SA, this microsensors system can be developed to simultaneously detect IAA and SA. It is necessary to emphasize that the sensitivity of IAA or SA detection is better using this microsensor than our previous constructed flat electrodes [25,26,27].

Continuous detection of IAA or SA was achieved using amperometric I-t curves recorded at 0.5 V or 0.75 V, respectively, on the electrochemical microsensor. The current values elevated steadily with the increasing concentration of IAA or SA with ascending concentrations of IAA (Figure 2C) or SA (Figure 3C) in PBS (0.2 M). A linear correlation was also found between current variation and the content of IAA (Figure 2D) or SA (Figure 3D), with IAA or SA contents ranging from 0.1 to 10 µM or 0.1 to 30 µM, respectively. The standard curve of continuous detection of IAA or SA was based on the change in the response current of the amperometric I-t curves, which laid a foundation for the subsequent continuous detection of IAA or SA in the plant samples.

**Figure 4:**
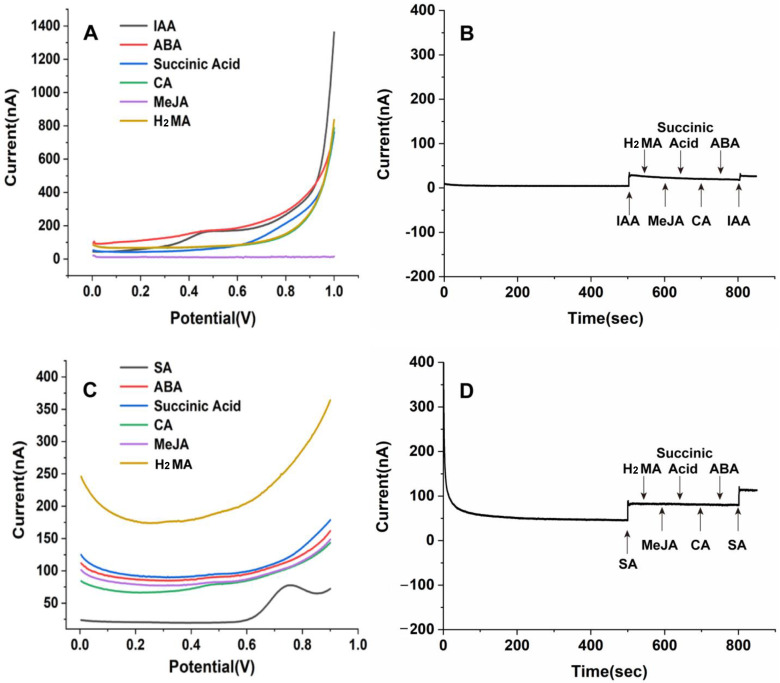
The selection of the Pt wire-based electrochemical microsensor for detection of IAA and SA. (**A**) DPV curves of 10 µM in IAA, malic acid (H_2_MA), methyl jasmonate (MeJA), citric acid (CA), succinic acid, and abscisic acid (ABA) on Pt wire-based electrochemical microsensor. (**B**) Amperometric response to continuous additions of 10 µL IAA, H_2_MA, MeJA, CA, succinic acid, ABA and IAA with concentration 10 mM, respectively, in 10 mL 0.2 M PBS with pH 7 at 0.5 V application potential. (**C**) DPV curves of 10 µM SA, H_2_MA, MeJA, CA, succinic acid, and ABA on Pt wire-based electrochemical microsensor. (**D**) Amperometric response to continuous additions of 10µL SA, H_2_MA, MeJA, Sussiinc Acid, CA, ABA, and SA with concentration 10 mM, respectively, in 10 mL solution with 0.2 M PBS (pH 7) at 0.75 V application potential.

To evaluate the reproducibility of the electrochemical microsensor, ten different working electrodes were tested using DPV and amperometric I-t. The findings demonstrated that the relative standard deviation (RSD) was approximately 3.25% and 3.75% for 10 µM IAA and SA, respectively, based on DPV, and 3.49% and 4.77% based on amperometric I-t, indicating good reproducibility for IAA and SA detection. In addition, potential interferences from other plant molecules in the detection of IAA (Figure 4A) and SA (Figure 4C) were investigated. No significant interferences were observed in detecting either IAA or SA from malic acid (H_2_MA), citric acid (CA), methyl jasmonate (MeJA), abscisic acid (ABA), or succinic acid at a concentration of 10 µM. The interferences from these molecules in the detection of IAA (Figure 4B) and SA (Figure 4D) were also evaluated using amperometric I-t, and no interference was found in detecting either IAA or SA from H_2_MA, MeJA, succinic acid, CA, or ABA at a concentration of 10 µM. Based on these results, the conducting carbon cement/MWCNTS/modified Pt wire-based microsensor was used to detect IAA and SA in real samples that also contain other plant molecules.

### 3.3. Continuous In Vivo Electrochemical Monitoring of IAA and SA in the Veinsof Tomato Leaves

High salinity is one of the most serious abiotic stresses for plants; it can cause great crop yield loss through its detrimental effect on plant growth and development. Phytohormones play key roles in plant response and adaptation to high salinity [30,31,32,33,34]. The adjustments of hormone levels in plants are the initial process to respond to high salinity stress [33]. IAA and SA are also associated with the response to high salinity stress [34,35,36,37,38,39,40]. As auxin receptor genes, the expression of *TIR2* and *AFB2* was inhibited by high salinity stress, which indicated that both of them can reduce the accumulation of the auxin under the high salinity stress [37]. In addition, the response of the tir1/afb2/afb3 mutant to high salinity stress was more sensitive, which showed that root growth slowed, indicating that the adaptation mechanism of plants to high salinity stress through the regulation of the auxin signal was activated [35]. The photosynthesis and antioxidant activity of plants under high salinity stress can be improved by the application of an SA treatment [38]. SA also can enhance the AM symbiosis and nitrogen fixation to reduce the harm caused by high salinity stress on plants [39]. The snc1 and sid2 (the deletion mutant of SA synthesis), which lead to the low accumulation of SA in plants, are more sensitive to high salinity stress [40,41].

**Figure 5:**
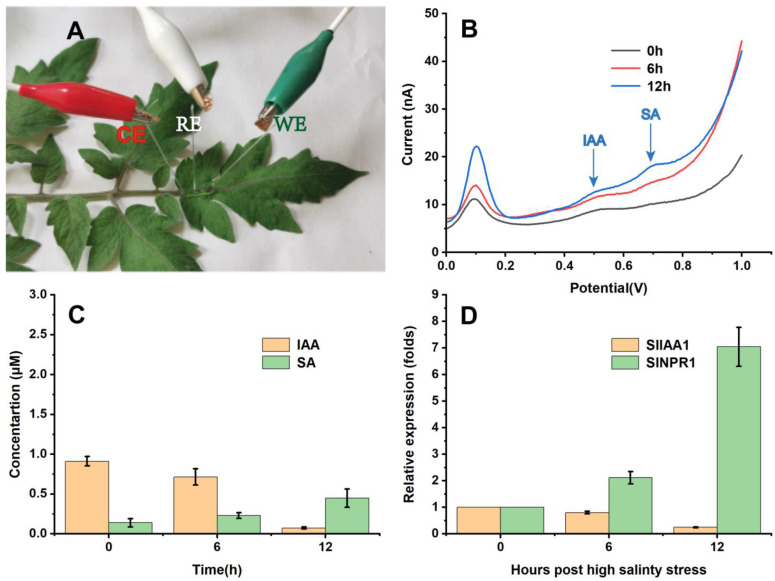
The MWCNTS/CCC/Pt wire-based electrochemical microsensor was used to identify IAA and SA contents in tomato leaf veins in situ. (**A**) Real image of the modified Pt wire-based electrochemical microsensor adopted for detecting tomato leaf vein IAA and SA in situ. (**B**) DPV curves for IAA and SA in tomato leaf veins at 0, 6, or 12 h after high salinity stress. (**C**) IAA and SA levels in tomato leaf veins at 0, 6, or 12 h after high salinity stress (*n* = 10), (**D**) Expressions of *SlIAA1* and *SlNPR1* in tomato leaf samples at 0, 6, or 12 h after high salinity stress (*n* = 10).

To study the crosstalk between IAA and SA in plants under high salinity stress, the electrochemical microsensor was used to monitor them in the veins of tomato leaves in situ. Figure 5 presents IAA and SA electrochemical monitoring results in tomato leaf veins using the microsensor. The microsensor consisted of a needle-like MWCNTS/CCC/Pt wire-based electrode, a needle-like Ag/AgCl electrode, and a needle-like Pt electrode that were inserted into tomato leaf veins. It should be emphasized that only 2 mm electrode tips were inserted into the veins of the tomato leaf. Subsequently, 10 µl PBS (0.2 M pH 7.0) was applied to the electrode surface to facilitate the electrochemical measurements. Comparing these sensors with the flat electrodes we had previously constructed to detect the IAA or/and SA either in situ or through micro-sampling [25,26,27], the sensors in this study caused significantly less damage to the plant.

Figure 5B depicts the DPV curves for IAA and SA in tomato leaf veins under high salinity stress. Compared to the starting point (0 h), the peak response of IAA was gradually decreasing while SA was increasing in the veins of tomato under high salinity stress at 6 and 12 h, respectively. Figure 5C shows the changes in IAA and SA contents in the veins of tomato. There was no obvious change at 6 h, but a significant reduction at 12 h for IAA was observed. On the contrary, the SA content showed an increasing trend and reached significant increases at 12 h. We also analyzed the expression pattern of IAA and SA related genes’, including *SlIAA1* and *SlNPR1*. *SlIAA1* is an auxin response gene induced by IAA [41], while *SlNPR1* makes a vital impact on SA signaling and is upregulated by SA [42,43]. Thus, *SlIAA1* and *SlNPR1* genes were used to confirm the detection results obtained in this study. Figure 5D reveals that the expression of *SlIAA1* in tomato leaf veins decreased significantly under high salinity stress relative to the control (0 h), while the expression of *SlNPR1* increased markedly under high salinity stress. These findings suggest that IAA levels were reduced while SA levels were elevated in tomato leaf veins under the high salinity stress. In addition, these change information of IAA and SA in tomato leaves can be used to reflect the health stage of tomato under the high salinity stress.

**Figure 6:**
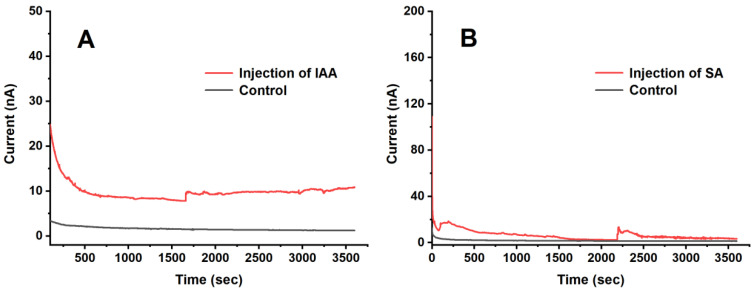
(**A**) The I-t detection curves of 10 µM IAA injected into the veins of tomato leaves at 1600 s (red line) and control (black line) (**B**) The I-t detection curves of 10 µM SA injected into the veins of tomato leaves at 1600 s (red line) and control (black line).

Until now, there has been no good method to reflect the dynamic changes of IAA and SA in plants. To investigate the dynamics of SA and IAA in tomato leaf veins, continuous monitoring of their levels was performed using a microsensor based on an amperometric I-t approach. Figure 6A,B shows the injection of 10 µM of IAA or SA in the vein of tomato 5mm away from the microsensor at the time point of 1600 s. The group that received an injection of IAA or SA in the veins of tomato leaves exhibited a clearly increasing peak in the I-t curve compared to the control. Such results suggested that the microsensor can be used to monitor the dynamic variation of IAA and SA.

**Figure 7:**
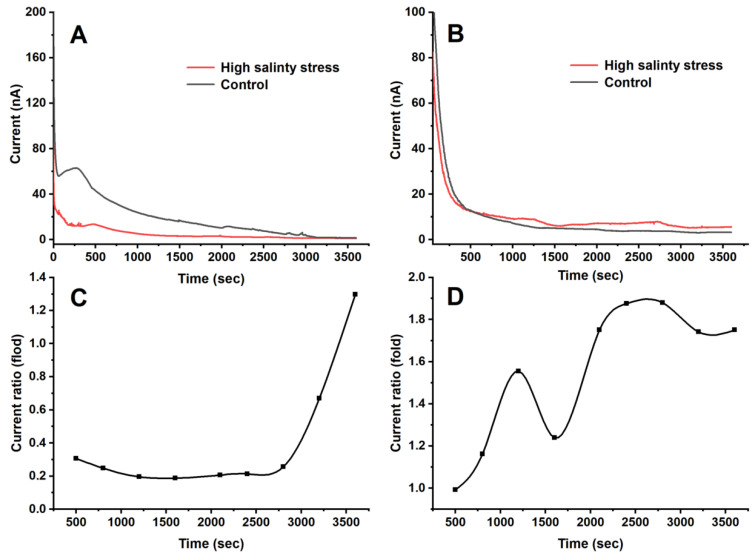
(**A**)The I-t detection curves of IAA in veins of tomato leaves under high salinity stress (red line) and control (black line) from 11.5 to 12.5 h. (**B**) The I-t detection curve of SA in veins of tomato leaves under high salinity stress (red line) and control (black line) from 11.5 to 12.5 h. (**C**) the current ratios of IAA in veins of tomato leaves under high salinity stress and the control (**D**) the current ratios of IAA in veins of tomato leaves under high salinity stress and the control.

Figure 7A,B presents the dynamics of IAA and SA in tomato leaf veins after high salinity stress from 11.5 to 12.5 h. For IAA, the curves under high salinity stress exhibited no fluctuations in the peaks compared to the control (Figure 7A). The current ratios for IAA in the veins of tomato leaves under high salinity stress and the control were also analyzed (Figure 7C). The results revealed that the current ratios for IAA were less than 0.3-fold during this period, indicating IAA suppression under high salinity stress. Meanwhile, for the SA group, the curves under high salinity stress exhibited multiple fluctuations in the peaks compared to the control (Figure 7B), and the current ratios for SA were higher than 1.5-fold during this period (Figure 7D), suggesting an increase in SA under high salinity stress. The current monitoring system allows (10 µL PBS) for continuous monitoring of IAA or SA within three hours. It should be emphasized that, if SA or IAA want to be monitored for a longer period of time, more PBS will need to be added to the surface of the electrode to ensure the conductivity.

## 4. Conclusions

IAA and SA play critical roles in regulating various physiological events in plants. The investigation of their regulatory mechanisms and crosstalk could be facilitated by the continuous in situ monitoring of SA and IAA dynamics. In the present study, a MWCNTS/CCC/Pt wire-based electrochemical microsensor was developed to constantly monitor IAA and SA in vivo. The dynamics of IAA and SA in tomato leaf veins after high salinity stress were monitored in vivo and constantly. Compared to conventional detection methods, this system can be used for in situ detection or continuous monitoring of IAA and SA with minimal damage to plant tissues. Importantly, this study represents the first report on the continuous monitoring of IAA or SA in vivo using an electrochemical microsensor. The continuous and dynamic changes in information about IAA and SA that are obtained using this system not only can help clarify the interaction mechanisms of IAA and SA in plants, it can also be used to evaluate the health status of plants. Overall, our proposed strategy is expected to not only shed light on basic research for the botany, but also for the development of precision agriculture.

## Figures and Tables

**Table 1:** The qRT-PCR primers used in this study.

Gene Name	Primer Set	
	Forward Primer (5′-3′)	Reverse Primer (5′-3′)
SLIAA1	ATGATGTTTTTCCTGTTAGATCTCACT	TGAATCTAAGTCAAGTTCTGATCATGTC
SLNPR1	GGGAAAGATAGCAGCACG	GTCCACACAAACACACACATC
β-actin	GTCCTCTTCCAGCCATCCAT	ACCACTGAGCACAATGTTACCG

## Data Availability

Data are contained within the article.
